# Increasing sustainability in pork production by using high inclusion levels of co-products distillers dried grains with solubles, wheat middling and canola meal doesn’t affect pig growth performance and meat quality but reduces boar taint

**DOI:** 10.5713/ab.22.0468

**Published:** 2023-02-27

**Authors:** Thanh T. Nguyen, Shola G. Olumodeji, Kirsty L. Chidgey, Timothy J. Wester, Carolina E. Realini, Patrick C. H. Morel

**Affiliations:** 1 Monogastric Research Centre, School of Agriculture and Environment, Massey University, Palmerston North 4442, New Zealand; 2 The University of Danang, Campus in Kon Tum, 704 Phan Dinh Phung, Kon Tum 60107, Vietnam; 3 School of Agriculture and Food Science, University College Dublin, Belfield, Dublin 4 VIW8, Ireland; 4 AgResearch Ltd, Te Ohu Rangahau Kai, Turitea 4474, New Zealand

**Keywords:** Canola Meal, Distillers Dried Grains with Solubles (DDGS), Indole, Pig Production, Skatole, Wheat Middling

## Abstract

**Objective:**

The present study is to examine the effect of high inclusion of co-products in pig diets (referred to as an alternative diet) during the finishing stage on pig growth performance, meat quality and boar taint compounds.

**Methods:**

Growing pigs were fed an alternative diet made with distillers dried grains with solubles (25%), canola meal (20%), and wheat middling (15%) or a control diet based on barley and soybean meal to investigate the impact of co-products on pig performance and meat quality. Sixteen female and sixteen entire male Duroc×(Large White×Landrace) pigs (22.6±2.07 kg, body weight±standard error) were equally allocated to the diets.

**Results:**

Pigs fed the alternative diet had a lower feed intake; however, growth rate and feed conversion efficiency were unaffected by diet. A diet by sex interaction was found for gain:feed whereby males fed the alternative diet had the best feed conversion (p<0.01). Pork from pigs fed the alternative diet had lower a* and Chroma and protein % (p<0.05), while other meat quality characteristics were unaffected. The alternative diet reduced backfat skatole levels (p<0.001).

**Conclusion:**

A diet containing high inclusion levels of co-products can be fed to pigs during the finishing stage without detrimental effects on pig performance or meat quality and with the potential to enhance pork flavour. This finding suggests a solution to increase the sustainable development of pig production.

## INTRODUCTION

Pig meat is the second most commonly consumed meat in the human diet and production is projected to grow to meet the demand of an increasing human population [1]. Nonetheless, feeding pigs with conventional feedstuffs, which are primarily composed of cereal grains and soybean meal (SBM), is unsustainable for the development of the pig industry. Pigs compete directly with human food sources, arable land and agricultural resources [2]. Furthermore, the expansion of soybean production is closely linked to deforestation, biodiversity loss, and greenhouse gas emissions [3]. On the other hand, as feed accounts for approximately 60% to 70% of the direct cost of pig production, reliance on volatile and competitive commodity markets to supply feed can compromise economic returns.

It is possible to replace conventional feedstuffs in pig diets, given that they are omnivores and are therefore suited to ingest many types of feed. Substituting conventional feed ingredients with co-products in pig diets is a key strategy for making pig production more sustainable [4]. Distillers dried grains with solubles (DDGS), canola meal (CM) and wheat middling (WM) are co-products from biofuel and food processing that hold potential as feedstuffs for pigs. They can be included in grower -finisher diets for pigs, however, need a proper risk management as they contain high levels anti-nutritional factors such as insoluble fiber, which limits their use for non-ruminants [4]. The inclusion of DDGS, CM and co-extruded full-fat flax seed and field pea can be up to 50% in grower-finisher diets [5]. Compared with growing pigs, finishing pigs have greater gut capacity which possibly removes the physical limitation to digesting high fiber diets. Therefore, the present study is expected to maximize the inclusion of DDGS, CM, and WM above the previous findings in finishing pig diets with or without negligible depletion in pig growth performance, carcass yield, and meat quality.

## MATERIAL AND METHODS

The experiment was conducted at the Massey University Pig Biology Unit, Palmerston North, New Zealand and was approved by the Massey University Animal Ethics Committee (MUAEC 19/125).

### Experimental diets

A control diet and an alternative finisher diet (alternative diet) were used in the study (Table 1). The main ingredients of the control diet were barley, SBM and soybean oil, while the main ingredients of the alternative diet were co-products: DDGS, CM, and WM. The nutrient composition of both diets was formulated to meet or exceed requirements of growing-finishing pigs according to NRC [6]. The control and alternative diets were equal in digestible energy and apparent ileal digestibility of lysine. Both diets were pelleted.

### Animals and handling

Sixteen female and sixteen entire male Duroc×(Large White× Landrace) pigs from a commercial farm were used for this experiment. The pigs were weighed upon arrival (22.6±2.07 kg, average±standard deviation) and allocated to 4 pens. Two pens housed female pigs, and the other two pens housed male pigs. All pigs were fed the control diet for 7 weeks during the growing phase. Subsequently, for the finishing period of 3 weeks, female and male pigs from 2 pens continued with the control diet while the female and male pigs from the other 2 pens were fed the alternative diet. Each pig was identified with a numbered tag in the left ear and a radio frequency identification tag in the right ear. Pigs had *ad libitum* access to food and water throughout the experiment. Individual feed intake was recorded by electronic feeders and the experimental unit was the individual pig. One female pig allocated to the alternative finishing group was removed from the trial in week 2 due to diarrhea. Data from this pig was excluded from the analysis.

All pigs were housed in one building and grouped in pens measuring 20 m2 with a solid concrete floor, enabling a space allowance of 2.5 m2/pig. Each pen was equipped with a nipple water drinker and a single-space electronic feeder (F.I.R.E. feeder; Osborne Industries, Inc., Osborne, KS, USA). The feeder was calibrated once a week. Each pen had a sleeping area separated from the main pen by a wall and accessed via a doorway. Feed and water sources were located in the main pen. Pigs had unlimited access to all areas of their pens. Temperature and airflow were managed with heat lamps (in the sleeping areas) and mechanical ventilation. The main room temperature was maintained between 18°C to 21°C.

Feed intake (g of feed per visit per pig) was automatically recorded by the F.I.R.E. feeder system. The feeder entrance was covered by an adjustable protective race so that only one pig could eat at a time. Frequency, time, and duration of feeding as well as feed intake were recorded daily for each pig. Intakes less than 0 and greater than 2,000 g per visit were disregarded as errors.

The live weight of each pig was recorded weekly. Individual average daily gain, daily feed intake, and gain:feed were calculated for each experimental period (weeks 1 to 7 and 8 to 10) and the entire experiment (weeks 1 to 10). Feed was sampled daily, pooled, and stored at 4°C before analysis.

### Slaughter

The pigs were transported for approximately two hours to a commercial abattoir (Land Meat Ltd, Wanganui), rested overnight, and slaughtered the next morning. Hot carcass weight without kidneys and leaf fat, and back fat depth (BFD) were recorded within 30 minutes post-slaughter. BFD was measured in the right side of the carcass at the P2 position, about 65 mm from the dorsal mid-line at the level of the last rib, using a Hennessy grading probe (Hennessy Technology, Auckland, New Zealand).

The following day the carcasses were cut, and the bone-in loins were vacuum packaged and transported to Massey University and stored frozen (−20°C) until further meat quality analysis.

### Meat quality

The bone-in loin (*M. longissimus thoracis*.) was defrosted at 4°C over 48-hours and was removed from the bone with the subcutaneous fat left on. The loin was subdivided into 4 portions. A 4 cm section of the cranial portion was used to measure pH and loin chemical composition. A 4 cm section in the mid portion was used to assess drip loss. The next two 2.5 cm wide sections in the mid loin were used for cooking loss and shear force measurements.

#### pH

The pH was measured at 45 minutes (pH45) after slaughter in the loin muscle at P2 by a pH spear adjusted by temperature (OAKTON, EUTECH Instruments, Vernon Hills, IL, USA). The ultimate pH was measured after thawing at three points from medial to distal across a transverse, internal cut of the striploin with a pH spear adjusted by temperature (Eutech Instruments, Singapore). The pH spear was calibrated to pH 4.01, 7.00, and 10.01 standard buffers.

#### Colour

The lean meat colour was measured on a fresh cut, transverse surface after 1-hour blooming time using the Minolta Colour Meter calibrated to a standard white tile supplied by the manufacturer (CR-200; Konica Minolta Photo Imaging Inc., Mahwah, NJ, USA). The CIE L* (lightness), a* (redness), and b* (yellowness) values were measured. Chroma C* and hue angle h° were calculated using the below equations:


$$\text{Chroma}=\sqrt{a{*}^{2}+b{*}^{2}}\;\text{and}\;\text{Hue}=\text{arctan}\;\frac{b^{*}}{a^{*}}$$

#### Drip loss

Two cubes of raw meat with 4 cm sides were cut from the 4 cm steak. The 4 cm cube was weighed then suspended on a net in a plastic bag at 4°C. After 48 h, the suspended cube was blotted dry using tissue paper and reweighed. Drip loss was calculated as the original weight minus the weight at 48 h and the value was expressed as a percentage of the original weight. The value of drip loss of an animal was the mean of the drip loss from two cubes.

#### Cooking loss

Meat was cooked in three batches and samples were allocated randomly with the condition that all treatments were equally represented in each batch. The 2.5 cm steaks were weighed and then suspended in vacuum bag in a water bath and cooked at 70°C for 60 min. Fluid from the bag was poured off and the samples were left to cool at 2°C for 24 h. Steaks were then removed from the bag, blotted dry, and re-weighed. Cooking loss was calculated as the difference in weight of the two 2.5 cm steaks before and after cooking and expressed as a percentage of the weight before cooking. The value of cooking loss of an animal was the mean of the cooking loss from two steaks taken from that animal.

#### Shear force

Round cores (diameter = 1.27 cm) were removed parallel to the longitudinal orientation of the muscle fibers from each steak. Shear force measurements were determined using a texture analyzer (Stable Micro System; TA.HD Plus texture analyzer, Surry, UK) fitted with a Warner-Bratzler shearing blade with a set crosshead speed at 200 mm/min. The samples were sheared perpendicular to muscle fiber orientation. A maximum of 8 cores were obtained from both steaks per animal and the mean shear force values were reported in Newtons.

#### Loin chemical composition

The portion used for pH then was finely minced (Kenwood MG450, 3 mm hole-plate), vacuum-packed and frozen until assessing chemical composition.

### Chemical analyses

Feed samples were pooled by diet and stored at 4°C during the experiment and meat samples were stored at −20°C until chemical analyses at the Massey University Nutrition Laboratory, Palmerston North, New Zealand. Gross energy (GE) of the trial diets was determined by combusting the sample completely in a bomb calorimeter (AC-350; LECO Corporation, St. Joseph, MI, USA). Other components were analyzed according to the method of AOAC: dry matter (AOAC 925.10 and 930.16); crude protein (AOAC 968.06, Dumas method); fat (AOAC 922.06, Mojonnier method,); crude fiber (AOAC 962.09/978.10 - modified); neutral detergent fibre (NDF) (Fibretec, Foss, Hoganes, Sweden; AOAC 2002.04); acid detergent fibre (ADF) (Fibretec, Sweden; AOAC 973.18); lignin (Fibertec, Sweden; AOAC 973.18); starch (α-amylase Megazyme kit; Megazyme International Ireland, Bray, County Wicklow, Ireland; AOAC 996.11); ash (furnace 550°C, AOAC 942.05); calcium (preparation AOAC 968.08D followed by colorimetric analysis); phosphorus (preparation AOAC 968.08D, ISO6491.1998E, modified in-house method); amino acid profile (acid stable: HCl hydrolysis followed by RP HPLC separation using AccQ Tag derivatization, AOAC 994.12); cysteine/methionine (performic acid oxidation, AOAC 994.12); tryptophan (AOAC 2017.03, sub-contracted, non-accredited); acid insoluble ash (acid reflux ash, non-accredited method).

Loin and backfat samples were stored at −20°C until analysis. Chemical composition of meat was analyzed by the methods: AOAC 950.46B for dry matter; furnace 550°C AOAC 920.153, 923.03 for ash; Soxtec, AOAC 991.36 for fat and AOAC 968.06 (Dumas method) for crude protein. Androstenone, indole and skatole concentrations in back fat samples were determined following the method of Fischer et al [7].

### Statistical analysis

All statistical analyses were performed using the SAS software, version 9.4 TS level 1.6 (SAS Institute Inc., Cary, NC, USA). A linear model (Proc general linear model) with pig diet, sex, and their interaction as fixed effects was fitted to the growth performance, carcass characteristics, meat quality attributes and boar taint indicators. In addition, cooking batch was included as a random effect in the model for the analysis of meat cooking loss and shear force variables. Initial live weight was initially added in the model as a covariate but was removed as it was not significant.

## RESULTS

### Chemical composition of the diets

The chemical composition differed between the control and alternative finishing diets (Table 2). Crude protein, fat, and crude fiber contents were higher in the alternative finishing diet. Lignin and ADF contents in the alternative finishing diet were more than double that of the control diet. Conversely, the alternative finishing diet contained less starch compared to the control diet. Dry matter, GE, and ash concentrations in the alternative finishing diet were slightly higher than in the control diet. In addition, the alternative diet had a higher essential amino acid content than the control diet, except for phenylalanine.

### Growth performance and carcass traits

Pig growth performance and carcass yield are presented in Table 3. Replacing the control diet with the alternative diet in the finishing stage reduced feed intake (p<0.05) but did not impair growth rate or feed conversion efficiency (p>0.05) of finishing pigs. Although pigs in the alternative finishing diet treatment had a lower gain:feed, lower initial live weight and lower finishing live weight for the 7-week growing period (p<0.05), no differences for these parameters were found between treatment groups for the entire 10 weeks of the experiment. When the initial live weight was included in the model as a covariate, the results did not change.

Sex influenced growing-finishing pig performance. Feed conversion of male pigs was more efficient than in females (p<0.05) in all phases of the experiment. Male pigs also grew faster than females in the finishing stage (1,077 vs 963 g/d for males and females, respectively). However, growth rate in the growing phase and overall experiment was similar between male and female pigs.

The only significant interaction between diet and sex was observed for gain:feed in the finishing stage (p<0.01). Post-hoc analysis (not presented in the table) indicated that males fed the alternative finishing diet tended to have the greatest gain:feed (0.42), while female pigs fed the same diet tended to have the lowest gain:feed (0.35). Equivalent results were obtained if the initial weight of the finishing phase was included as a covariate.

Carcass weight, dressing out percentage, and BFD were not different between treatment groups or sex. However, pigs fed the control diet tended to have a heavier carcass weight (p = 0.061) and higher dressing percentage (p = 0.093) than pigs fed the alternative finishing diet.

### Meat quality and boar taint

Pork quality characteristics of loins and boar taint of backfat from male and female pigs fed the two diets were displayed in Tables 4 and 5, respectively.

There were no interactions (p>0.05) between diet and sex for the meat quality variables measured, except for cooking loss (p<0.05). Neither dietary treatment nor sex influenced ultimate pH, drip loss at 48 h, shear force, or hue angle (Table 4). However, meat from intact male pigs had a higher pH45 than that of female pigs (p<0.05). Cooking loss of meat from males was higher than females when fed the alternative diet (30.42 vs 27.81; p<0.05), but no differences (p>0.05) were found in cooking loss due to sex when pigs were fed the control diet. Meat from pigs fed the alternative finishing diet had lower a* and Chroma color values than meat from pigs fed the control diet (p<0.05). Intact male pigs had meat that was lighter and more yellow and had a higher Chroma value than that from gilts (p<0.05).

Dietary treatment did not affect the chemical composition of the loin muscle, except for crude protein concentration which was slightly greater in muscle of pigs fed the control diet (p<0.05). There were no differences in dry matter, crude protein, or ash between meat from female or male pigs (p> 0.05).

There was no diet×sex interaction (p>0.05) for compound indicators of boar taint in the adipose tissue (Table 5). Pigs fed the alternative finishing diet had half the skatole concentration in adipose tissue than those fed the control diet (p< 0.001). Concentrations of androstenone, indole and skatole in adipose tissue were all greater in male compared to female pigs (p<0.05).

## DISCUSSION

This paper aims to examine the effects of high inclusion levels of co-products in finishing diets on pig growth performance, meat quality, and boar taint. We focus on discussing the effect of co-product inclusion and not the impact of sex on these parameters, as previous studies have already revealed the impact of sex on these factors. The main findings of the present study indicate that a three-week feeding of the co-product diet did not have a significant impact on growth performance and meat quality. Moreover, we observed a reduction in skatole levels in the backfat of pork fed with the high inclusion of co-product in their diet. These findings are considered important in the field of pig nutrition and production.

### Pig growth performance and carcass yield

The major impact of the alternative diet on pig growth performance in this experiment was for feed intake, though this effect was not unexpected. High fiber and antinutritive factors in the alternative diet could explain the significantly lower feed intake of the pigs fed the alternative diet. Co-products like DDGS, CM, and WM are high in fiber content. In the present study, NDF, ADF, and lignin in the alternative finishing diet were approximately twice that of the control diet. Greater bulk volume of the alternative finishing diet might cause earlier satiety and then limit feed consumption [8]. On the other hand, a lower feed intake of the co-product diet can be caused by the presence of antinutritional factors like glucosinolates in CM, which are known to inhibit intake [9].

Nonetheless, a lower feed intake did not negatively influence pig growth rate, gain:feed or carcass yield of pigs fed the alternative diet. This finding is in agreement with many previous studies including those that used DDGS, CM, WM in growing-finishing pig diets. DDGS could be included up to 30% in growing–finishing pig diets without negatively affecting growth performance or carcass characteristics, providing that the diets were formulated with similar levels of standardized ileal digestible lysine and energy [10]. Replacing SBM with CM had no negative impact on pig growth performance. Inclusion of 24% solvent CM or 29.2% expelled CM in growing pig diets did not impair dry matter intake, feed conversion efficiency or liveweight gain [11]. Wheat middling can be included at up to 30% without impairing weight gain [12].

Co-products can be included together to maximize co-product inclusion in pig diets. Smit et al [13] demonstrated that feeding a diet of up to 240 g CM per kg and 150 g DDGS per kg to growing pigs had small effects on overall growth performance, and no impairment on carcass traits. The inclusion of DDGS, CM, and co-extruded full-fat flax seed and field pea can be up to 50% in grower-finisher diets [5]. Compared with growing pigs, finishing pigs have greater gut capacity which possibly removes the physical limitation to digesting high fiber diets. Based on previous studies, it was expected that a diet formulated with a combination of co-products to meet the nutrient recommendations for finishing pigs would have no effect on growth performance and carcass yield. In the present study, despite over half of the barley and all the SBM being replaced by alternative ingredients in the finishing phase, no negative effects on pig growth performance or carcass yield were observed.

In contrast, several studies indicated that DDGS should not be included in pig diets at levels above 20%, and that CM is not an effective replacement for SBM in grower and finisher pig diets [14,15]. It is important to note that these findings may vary depending on the specific source and processing of the DDGS, and CM being used [4]. Additionally, the length of the feeding period in a study can also impact the results, as the adaptation of pigs to a high-fiber diet can take several weeks [16]. In the present research, we fed the co-product diet for 3 weeks. The feeding period might not have been long enough for the alternative diet to show the impact of its high fiber content on growth. To our knowledge, there has been no research where over half of the conventional ingredients were substituted with co-products in grower-finisher pig diets. Our results show that the effect of a short-term inclusion of co-products at their maximum inclusion level did improve pork flavor (see the discussion on boar taint) without affecting of growth performance. However, further studies with longer feeding periods are needed to fully understand the effects on pig growth and performance.

### Meat quality

A combination of high fat and a low digestible carbohydrate content in an alternative diet may reduce muscle glycogen levels at the time of slaughter, which might increase muscle pH and water-holding capacity of pork [17]. However, the results in the present study did not show differences in ultimate pH or water-holding capacity (measured as drip loss at 48 hours) between the two diets.

Other studies using similar co-products in pig diets found no compromise in meat quality. For instance, loin muscle harvested from pigs fed diets containing levels of DDGS at 30% or 45% did not differ in marbling, color lightness (L), redness (a*), drip loss, tenderness, juiciness, or off-flavor characteristics, though the diet leads to softer bellies, higher polyunsaturated fatty acids levels in carcass fat, and higher iodine values [18]. Completely replacing SBM with other plant protein sources did not affect any pig meat quality parameters, including loin chemical composition [19]. In our study, the pigs fed the control diet in the finishing stage tended to be heavier than those fed the alternative diet, resulting in a slightly higher percentage of protein in the loin muscle. Loin protein tends to increase when slaughter weight increases [20]. Furthermore, the lower protein content in pork from pigs fed the alternative diet compared to the control diet might be due to slight differences in ideal protein balance. Although the crude protein content of the alternative diet was higher than that of the control diet (186 vs 159 g/kg), the feed intake of the former was lower than the latter. Based on the calculated diet composition, the daily ileal digestible ideal protein balance intake was higher for the control diet than the alternative diet (336 g/d vs 308 g/d, respectively). This may explain the difference in muscle protein content between the diets.

### Boar taint

An additional quality attribute that influences consumer acceptability is flavor, off-odours and off-flavor of pork. Pork sensory attributes like sweaty, musky, urine- or faecal-like odors and flavors are mainly associated with boar taint. It results from the accumulation of androstenone, skatole, and other indoles in fat tissues. While androstenone is produced in the testes, skatole and indole are formed by bacterial breakdown of tryptophan in the large intestine [21].

The alternative diet significantly reduced the skatole level in the backfat of male and female pigs. This finding contradicts the hypothesis that high dietary fiber in pig diets leads to less tryptophan being digested in the small intestine [22], hence, more tryptophan is available in the hindgut for bacteria to produce skatole. Based on NRC (2012)[6], we estimated that the ileal undigested tryptophan reaching the hindgut of the pig fed the control diet and the alternative diet were 0.42 and 0.53 (g/kg feed intake) respectively. Therefore, our findings support the hypothesis that high dietary fiber decreases skatole concentration in pork due to the availability of fiber for hindgut bacteria digestion. Firstly, dietary fiber encourages carbohydrate-fermenting bacteria population growth in the hindgut which both directly and indirectly affect skatole production. Undigested protein and tryptophan available in hindgut are utilized for the growing biomass of carbohydrate fermenting bacteria, resulting in less tryptophan availability for degradation into indolic compounds [23]. On the other hand, a decreased pH in the hindgut environment caused by an increase in short-chain fatty acids production from carbohydrate-fermentation inhibits skatole-producing bacteria which is optimal at a neutral pH [24]. Secondly, insoluble fiber increases the volume and water binding capacity of fecal bulk. Therefore, skatole will be diluted in the large intestine [25]. Consequently, less skatole will be in contact with the intestinal wall and, as a result, skatole absorption is reduced. In our study, the concentration of NDF, ADF, and lignin in the alternative diet were greater than in the control diet.

Several studies have demonstrated the effect of high fiber diets on the production and absorption of skatole. Hansen et al [26] reported that a 25% inclusion of lupins in finisher pig diets significantly reduced skatole in blood and backfat of both males and females after 1 week. Similarly, Pauly et al [27] reported that feeding 30% raw potato starch to entire male pigs one week before slaughter reduced skatole levels in loin back fat but had no effect on androstenone levels. However, there are a few studies showing no effect of dietary fiber on boar taint levels in backfat. Study such as Hawe et al [28] showed that feeding 40% sugar beet pulp did not reduce the concentration of skatole in subcutaneous fat. Curry et al [29] reported including DDGS by 10% for pigs from 35 to 105 kg live weight did not reduce skatole but linearly increase the indole concentration in the carcasses of the pigs.

These contradictory results might be explained by the differences in types, amount and ratio of dietary fiber of the experimental diets. It is clear that soluble dietary fiber is highly fermentable, providing a substrate for carbohydrate fermentting bacteria. Conversely, insoluble dietary fiber increases the passage rate of digesta and faecal bulk, diluting hindgut contents. Recent investigations also showed the interaction of fiber types and other nutritional dietary factors on hindgut fermentation [30]. Our results might suggest that the combination of different co-products and conventional feedstuffs in the alternative diets may supply adequate dietary fiber level to prevent skatole formation in the hindgut. We estimated that, the amount of soluble fibre was similar in the two diets (37.6 vs 39.1 g/kg feed), however, the insoluble fiber in the alternative diet was almost double as in the control diet (115.9 vs 211.9 g/kg feed).

Reducing skatole in pig meat is critical to meeting the expectations and preferences of consumers. Leong et al [31] reported that the average skatole threshold that Singaporean consumers perceive is 28 ng/g fat. This value is higher than what was measured in pigs fed the alternative finishing diet or female pigs in the current study, but not for pigs fed the control diet or male pigs. It implies that consuming pork from pigs fed the control diet in the finishing phase would result in an unpleasant experience for Singaporean people, while meat from pigs fed the alternative finishing diet may not. Surgical castration of male piglets at a young age is a common method of preventing boar taint, however, is questionable from an animal welfare perspective and is not practiced in some countries. This finding suggests that feeding high fiber co-products for a short period of three weeks before slaughter may reduce skatole levels in adipose tissue. This is important for the sustainable production of high-quality pork, particularly for consumers in Far Eastern Asia.

The use of co-products in pig feed not only provides farmers with alternative options to reduce their dependence on traditional feedstuffs, but also offers the potential for improving pork flavor without facing public pressure due to animal welfare concerns. Furthermore, as a recent article has revealed, feeding pigs a diet that includes DDGS is more environmentally efficient than a traditional diet [32]. This implies that incorporating DDGS into the diet of entire male pigs can provide benefits in terms of animal welfare, the environment, and profitability. Using co-products including DDGS in the diet of entire male pigs for a short period of time might be a solution to achieve greater sustainability in compliance with the United Nations Sustainable Development Goal 12 on responsible consumption and production.

## CONCLUSION

A high inclusion of DDGS (25%), CM (20%), and WM (15%) can replace 60% of the barley and completely replace the soybean products in finishing pig diets without negatively affecting growth performance, carcass yield, and meat quality. Additionally, the co-product diet significantly reduced skatole levels in subcutaneous fat. Thus, the alternative finisher diet can be utilized on commercial pig farms to reduce the reliance on conventional feedstuffs without negative effects on pig growth performance or meat quality, and with potential benefits in terms of pork flavor. This research adds to a growing body of literature on the use of co-products in finisher diets and underscores the importance of continued research in this area. Furthermore, the present study provides valuable information for pig farmers looking to maximize the efficiency and sustainability of their operations.

## Figures and Tables

**Table 1:** Ingredient composition (as-fed basis) of the experimental diets

Items	Dietary	

	Control	Alternative
Ingredient (%, unless noted)		
Barley	78.83	32.05
SBM	16.00	-
CM	-	20.00
Soybean oil	1.00	-
Wheat middling	-	15.00
Tallow	-	3.50
DDGS	-	25.00
Lysine	0.25	0.5
Methionine	0.20	0.10
Threonine	0.20	0.20
Tryptophan	0.02	0.05
Premix pig grower^1)^	0.20	0.20
Dicalcium phosphate	3.00	1.00
Sodium hydro-phosphate	0.20	0.30
Salt	0.10	0.10
Limestone	-	2.00
Calculated values		
Digestible energy (MJ/kg)	13.47	13.52
Apparent ileal digestible lysine (g/kg)	8.84	8.82

SBM, soybean meal; CM, canola meal; DDGS, distillers dried grains with solubles.

1) Pig grower finisher premix low copper (Nutritech International, Auckland, New Zealand) provided the following (per kg diet, as fed): 7,000 IU of vitamin A, 1,500 IU of vitamin D_3_, 35 IU vitamin E, 2 mg of vitamin K, 1.5 mg of vitamin B_1_, 3 mg of vitamin B_2_, 2 mg of vitamin B_6_, 15 μg of vitamin B_12_, 11 mg of pantothenic acid, 15 mg of niacin, 20 μg of biotin, 0.25 mg of folic acid, 90 mg of choline, 80 mg of iron (sulfate), 30 mg of manganese (sulfate), 1 mg of cobalt (chloride), 0.3 mg of selenium (sodium selenite), 115 mg of zinc (oxide), 20 mg of copper (carbonate), and 1 mg of iodine (potassium iodate).

**Table 2:** Proximate composition and amino acid profile of the experimental diets (g/kg, as fed basis, unless noted)

Items	Dietary	

	Control	Alternative
Proximate composition		
Dry matter	871.8	886.3
Crude protein (N×6.25)	158.8	185.6
Fat	35.9	77.4
Crude fiber	41.9	59.5
Neutral detergent fibre	144.3	246.2
Acid detergent fibre	45.5	89.6
Lignin	8.2	29.1
Starch	357.2	221.5
Ash	48.9	62.3
Ca	6.6	9.2
P	8.5	8.1
Gross energy (MJ/kg)	15.6	16.8
Amino acid profile		
Lysine	8.5	10.5
Threonine	6.6	8.4
Tryptophan	1.9	2.3
Methionine	4	4
Cysteine	2.9	3.8
Isoleucine	5.8	6.2
Histidine	4.7	5.6
Valine	7.5	9.1
Arginine	8.9	10.2
Phenylalanine	8.2	7.9
Tyrosine	5.4	5.7
Aspartic acid	12.1	11.8
Serine	6.4	7.1
Glutamic acid	32.1	31.7
Proline	12.6	12.4
Glycine	5.8	8
Alanine	5.9	7.5
Leucine	10.5	11.4

**Table 3:** LSmeans for growth performance of male and female pigs fed two diets (control vs alternative)

Items	Diet (D)		Sex (S)		Pooled SE	p-value		
		
	Control (n = 16)	Alternative (n = 15)	Female (n = 15)	Male (n = 15)		D	S	D×S
Finishing phase								
LWf (kg)	84.94	79.76	81.14	83.56	2.123	0.105	0.437	0.582
ADG (g/d)	1,059	981	963	1,077	33.7	0.142	0.028	0.083
ADFI (g/d)	2.923	2.564	2.712	2.774	109.3	0.029	0.689	0.9
Gain:feed	0.37	0.38	0.36	0.39	0.008	0.112	0.007	0.004
Whole experiment								
ADG (g/d)	890	831	841	881	29.1	0.179	0.349	0.582
ADFI (g/d)	2,023	1,905	1,968	1,961	68.3	0.233	0.944	0.963
Gain:feed	0.44	0.44	0.43	0.45	0.005	0.639	0.006	0.13
Slaughter								
Carcass weight (kg)	64.84	59.84	61.91	62.78	1.779	0.061	0.751	0.473
Dressing out (%)	76.32	74.97	76.3	74.99	0.558	0.093	0.102	0.494
BFD (mm)	8.69	8.52	8.46	8.75	0.26	0.679	0.44	0.649

SE, standard error; LWf, live weight finish; ADG, average daily weight gain; ADFI, average daily feed intake; BFD, back fat depth.

**Table 4:** LSmeans for pork quality characteristics of loins from male and female pigs fed the two diets (control vs alternative)

Items	Diet (D)		Sex (S)		Pooled SE	p-value		
		
	Control (n = 16)	Alternative (n = 15)	Female (n = 15)	Male (n = 16)		D	aS	D×S
Meat quality parameters								
pH45	6.20	6.32	6.18	6.34	0.053	0.100	0.039	0.869
Ultimate pH	5.46	5.43	5.45	5.44	0.019	0.210	0.582	0.478
Drip loss 48 h (%)	8.66	9.67	8.90	9.43	0.596	0.230	0.538	0.879
Cooking loss (%)^1)^	28.25	29.12	28.01	29.35	0.558	0.1796	0.030	0.036
Shear force, N^1)^	55.30	53.64	56.52	52.42	2.841	0.610	0.180	0.312
Meat color								
L*	52.28	52.69	51.60	53.37	0.595	0.588	0.045	0.874
a*	6.49	5.51	5.72	6.27	0.279	0.021	0.173	0.970
b*	7.66	7.29	7.09	7.85	0.213	0.277	0.020	0.419
*C*	10.07	9.14	9.13	10.09	0.297	0.043	0.031	0.688
*H*	49.99	53.00	51.22	51.76	1.098	0.060	0.742	0.699
Chemical composition, % as fresh meat								
Dry matter	25.84	25.63	25.80	25.66	0.140	0.267	0.516	0.293
Crude protein	23.69	23.23	23.54	23.38	0.153	0.045	0.456	0.321
Fat	1.74	1.60	1.75	1.59	0.144	0.447	0.474	0.242
Ash	1.18	1.17	1.17	1.19	0.015	0.705	0.317	0.670

SE, standard error.

1) Adjusted for cooking batch effect.

**Table 5:** Boar taint compounds in back fat of male and female pigs fed two diets (control vs alternative)

Boar taint compounds (ng/g of fat)	Diet (D)		Sex (S)		Standard error	p-value		
		
	Control (n = 12)	Alternative (n = 12)	Female (n = 12)	Male (n = 12)		D	S	D×S
Indole	20.4	22.1	15.3	27.2	2.77	0.356	0.007	0.314
Skatole	35.8	18.2	23.3	30.7	2.48	<0.001	0.048	0.767
Androstenone	871	813	179	1504	110.2	0.303	<0.001	0.706
